# The Jak2 Small Molecule Inhibitor, G6, Reduces the Tumorigenic Potential of T98G Glioblastoma Cells *In Vitro* and *In Vivo*

**DOI:** 10.1371/journal.pone.0105568

**Published:** 2014-08-27

**Authors:** Rebekah Baskin, Sung O. Park, György M. Keserű, Kirpal S. Bisht, Heather L. Wamsley, Peter P. Sayeski

**Affiliations:** 1 Department of Physiology and Functional Genomics, University of Florida College of Medicine, Gainesville, FL, United States of America; 2 Research Centre for Natural Sciences, Hungarian Academy of Sciences, Budapest, Hungary; 3 Department of Chemistry, University of South Florida, Tampa, FL, United States of America; 4 Department of Physiological Sciences, University of Florida College of Veterinary Medicine, Gainesville, FL, United States of America; University of Pécs Medical School, Hungary

## Abstract

Glioblastoma multiforme (GBM) is the most common and the most aggressive form of primary brain tumor. Jak2 is a non-receptor tyrosine kinase that is involved in proliferative signaling through its association with various cell surface receptors. Hyperactive Jak2 signaling has been implicated in numerous hematological disorders as well as in various solid tumors including GBM. Our lab has developed a Jak2 small molecule inhibitor known as G6. It exhibits potent efficacy *in vitro* and in several *in vivo* models of Jak2-mediated hematological disease. Here, we hypothesized that G6 would inhibit the pathogenic growth of GBM cells expressing hyperactive Jak2. To test this, we screened several GBM cell lines and found that T98G cells express readily detectable levels of active Jak2. We found that G6 treatment of these cells reduced the phosphorylation of Jak2 and STAT3, in a dose-dependent manner. In addition, G6 treatment reduced the migratory potential, invasive potential, clonogenic growth potential, and overall viability of these cells. The effect of G6 was due to its direct suppression of Jak2 function and not via off-target kinases, as these effects were recapitulated in T98G cells that received Jak2 specific shRNA. G6 also significantly increased the levels of caspase-dependent apoptosis in T98G cells, when compared to cells that were treated with vehicle control. Lastly, when T98G cells were injected into nude mice, G6 treatment significantly reduced tumor volume and this was concomitant with significantly decreased levels of phospho-Jak2 and phospho-STAT3 within the tumors themselves. Furthermore, tumors harvested from mice that received G6 had significantly less vimentin protein levels when compared to tumors from mice that received vehicle control solution. Overall, these combined *in vitro* and *in vivo* results indicate that G6 may be a viable therapeutic option against GBM exhibiting hyperactivation of Jak2.

## Introduction

Glioblastoma mulitforme (GBM) is the most common and most aggressive form of primary brain tumor. The median survival is 14 months after optimal therapy such as surgical resection, radiation therapy, and/or chemotherapy. The most commonly used chemotherapeutic agent for GBM is temozolomide, which acts as a DNA alkylating agent. However, temozolomide resistance in a large number of GBM patients has prompted the development of alternate therapies [1]. Recently, some of the molecular mechanisms involved in GBM pathogenesis have been identified and these discoveries have led to the development of molecular targeted therapies. Pathways that have been targeted to date include VEGF, EGFR, PDGF, PI3K, Akt, and mTOR [2]. Although many of these therapies have shown promising pre-clinical efficacy, the clinical outcomes have not been highly successful thus far [3]–[4].

Vimentin is a type III intermediate filamentous protein. Along with actin and tubulin, it comprises the cytoskeleton of the cell and hence plays an important role in anchoring various organelles within the cytosol. It is highly expressed in mesenchymal cells and serves as an extremely reliable marker for indicating epithelial-to-mesenchymal transition [5]. Vimentin is overexpressed in a number of tumors including those of the brain, breast, lung, and prostate. Furthermore, within these cancers, vimentin expression correlates with accelerated tumor growth, increased metastatic potential, and poorer prognosis [6]. Within the brain, vimentin expression is observed in all grades of astrocytomas [7]. In addition, a recent report identified a positive correlation between glioma grade and vimentin expression and these same authors found that temozolomide resistance is associated with an up-regulation of vimentin [8]. When taken together, these results indicate that vimentin is both a marker of brain tumor pathogenesis and a predictor of chemotherapy resistance.

Recently, there has been increasing interest in the role of Jak/STAT signaling in GBM and the use of Jak/STAT small molecule inhibitors for the treatment of these tumors. Specifically, in 2007, constitutive phosphorylation of Jak2 was found in the GL15 glioblastoma cell line, and treatment with tyrphostin AG490, a pan tyrosine kinase inhibitor, was shown to induce cell cycle arrest in these cells [9]. More recently, studies have demonstrated the efficacy of more specific Jak2 kinase inhibitors in both cell culture and animal models of GBM [10], [11]. Along these lines of investigation, our laboratory has spent the past several years identifying Jak2 specific small molecule inhibitors. One compound in particular, G6, has shown exceptional *in vitro* and *ex vivo* therapeutic efficacy [12], [13]. In addition, it has been highly efficacious in three mouse models of Jak2-mediated hematological disease [14]–[16].

Here, we hypothesized that G6 treatment would reduce the tumorigenic potential of GBM cells that exhibit constitutive Jak2 signaling. To test this, we first screened GBM cell lines in order to identify those with increased levels of phospho-Jak2. We found that the T98G cell line expressed readily detectable levels of the protein and furthermore, the existing Jak2 was hyper-active. We found that G6 treatment of these cells significantly reduced the tumorigenic potential both *in vitro* and *in vivo* including significant reductions in vimentin protein levels within tumors that were harvested from G6 treated mice. When taken together, our data indicate Jak2 inhibitors in general, and G6 in particular, may be viable therapeutic options against GBM exhibiting constitutive Jak2 signaling.

## Materials and Methods

### Cell Lines

T98G glioblastoma cells were originally obtained from ATCC and kindly provided to us by Dr. Jeffrey Harrison (University of Florida). T98G cells were maintained in MEM supplemented with 10% FBS, 10 U/ml penicillin, 10 µg/ml streptomycin, and 2 mM L-glutamine at 37°C and 5% CO_2_.

### Cell Proliferation Assay

T98G cells were plated in 96-well plates and treated with the indicated concentrations of G6 for 72 hours. Cell viability was determined using the MTS colorimetric assay (Promega) according to the manufacturer's protocol.

### Colony Formation Assay

T98G cells were plated in 100 mm dishes at a density of 200 cells per dish and were cultured for 24 hours in the presence or absence of G6. The drug was then removed, and cells were cultured for an additional nine days. Cells were then washed with PBS, fixed in 90% methanol, stained with crystal violet, and the numbers of colonies were counted.

### Western Blotting

Cells were lysed in RIPA buffer (20 mM Tris pH 7.5, 10% glycerol, 1% Triton X-100, 1% deoxycholic acid, 0.1% SDS, 2.5 mM EDTA, 50 mM NaF, 10 mM Na_4_P_2_O_7_, 4 mM benzamidine, 1 mM phenylmethylsulfonyl fluoride, 1 mM Na_3_VO_4_, and 10 µg/mL aprotinin) and protein concentration was determined using a Bradford assay (BioRad). Approximately 30 µg of soluble protein was separated by SDS-PAGE and transferred onto nitrocellulose membranes. Membranes were blocked at room temperature for 1 hour with 5% milk in TBS-T and incubated with primary antibodies overnight at 4°C. The following primary antibodies were used: pY1007/p1008-Jak2 at 1∶300 (Invitrogen), total Jak2 at 1∶1000 (Millipore/Invitrogen), pY705-STAT3 at 1∶300 (Santa Cruz), total STAT3 at 1∶1000 (Santa Cruz), vimentin at 1∶2000 (Abcam), Caspase-3 at 1∶500 (Cell Signaling), and β-actin at 1∶500 (Cell Signaling). Secondary antibodies were applied for 1 hour at room temperature. Anti-rabbit and anti-mouse secondary antibodies were used at 1∶4000 (GE Healthcare) and bands were detected via reaction with an enhanced chemiluminescent substrate (Perkin Elmer).

### Cell Migration and Invasion Assays

For scratch assays, cells were plated on 4-well chamber slides, grown to confluency, and treated with G6 for 24 hours. The drug was then removed, a scratch was made, and migration into the scratch area was monitored 24 and 48 hours later. For cell migration assays, 5×10^4^ T98G cells were plated in 24-well migration inserts (PET membrane with 8 µm pores, BD Biosciences). Cells were plated in the top chamber in serum-free MEM and the bottom chamber was filled with serum-containing MEM. Cell migration was analyzed 24 hours later. Non-migrating cells were removed from the top of the chamber using a cotton swab, and migrated cells were stained with crystal violet and counted. The cells were counted at 10X magnification, using two random fields per chamber well. For cell invasion assays, 5×10^4^ T98G cells were plated in 24-well inserts coated with matrigel (BD Biosciences). The top chamber contained cells in serum-free MEM and the bottom chamber contained complete MEM. Cell invasion was analyzed after 48 hours. The cells were counted at 10X magnification, using two random fields per chamber well.

### Lentiviral shRNA-Mediated Jak2 Knockdown

HEK293T cells were transfected with Jak2 shRNA constructs (pool of Thermo catalog numbers RHS3979-9571807, RHS3979-9571808, RHS3979-9571809, RHS3979-9571810, and RHS3979-9571811) or a non-targeting shRNA construct (AddGene plasmid 1864) along with packaging and envelope vectors (psPAX2 and pMD2.G). Lipofectamine (Invitrogen) was used as the transfection reagent. Approximately 40 hours post-transfection, culture media containing virus was collected and supplemented with polybrene, sterile filtered, and applied to T98G cells. A second round of infection was performed 24 hours later. Infected cells were identified by puromycin selection and used for subsequent experiments.

### Apoptosis Assays

For TUNEL staining, cells were plated on 4-well chamber slides and treated with G6 for 48 hours. TUNEL staining was performed as previously described [17].

### T98G Xenografts in Nude Mice

All procedures were approved by the Institutional Animal Care and Use Committee at the University of Florida. T98G cells are known to have a relatively weak *in vivo* tumorigenic growth potential, even in immunocompromised animals [18], [19]. For this reason, 20 female athymic nude mice were flank injected with 5×10^6^ T98G cells in 150 µl of a 1∶1 mixture of RPMI media and Matrigel (BD Biosciences). Tumors were measured using an external caliper and volume was calculated by the formula 4π/3×(length/2)×(width/2)^2^ as previously described [18]. After 90 days of growth, only nine mice had tumors with a volume that was >50 mm^3^. These nine animals were randomly assigned to one of two groups; vehicle control (n = 4) or G6 (n = 5). The beginning tumor volumes for the two groups were 101+/−18 mm^3^ and 112+/−17 mm^3^, respectively. Mice then began receiving daily intra-peritoneal injections of either vehicle control solution (DMSO) or 10 mg/kg/day of G6 and tumor volumes were measured approximately every fifth day. After 35 consecutive days of treatment, the mice were euthanized and the tumors were resected for subsequent analyses.

### Apoptotic Gene Profile Analysis of Tumor Sections

RNA was isolated from each freshly harvested tumor using the RNeasy Mini Kit (QIAGEN). RNase-free DNase was used to eliminate DNA contamination. cDNA was synthesized from 2 µg RNA using the high capacity cDNA Reverse Transcription Kit (Applied Biosystems). Real-time PCR was carried out in a Multicolor Real-Time PCR Detection System using TaqMan gene expression assays (Applied Biosystems). All PCR amplifications were performed in triplicate. Parallel measurements of β-actin were also done as an internal control for normalization purposes.

### Histological Analysis of Tumors

Tumors were fixed in buffered formalin, paraffin embedded, and sectioned at 4 µm. Sections were then stained with either hematoxylin & eosin or Masson's trichrome. Images were examined for a number of characteristics including morphology, cellularity, and fibrosis. Tumor sections were also subjected to anti-phospho-Jak2, anti-phospho-STAT3, anti-Ki-67, and anti-vimentin immuno-histochemical (IHC) analyses. The numbers of positive stained cells were determined via the ScanScope software system (Aperio).

### Statistical Analysis

All results were expressed as mean +/− SEM. Statistical comparisons were done using Student's *t* test or a repeated-measures ANOVA for multiple comparisons. Data were considered to be significantly different when p<0.05.

## Results

### G6 Reduces Jak2 and STAT3 Phosphorylation in T98G Cells

**Figure 1:**
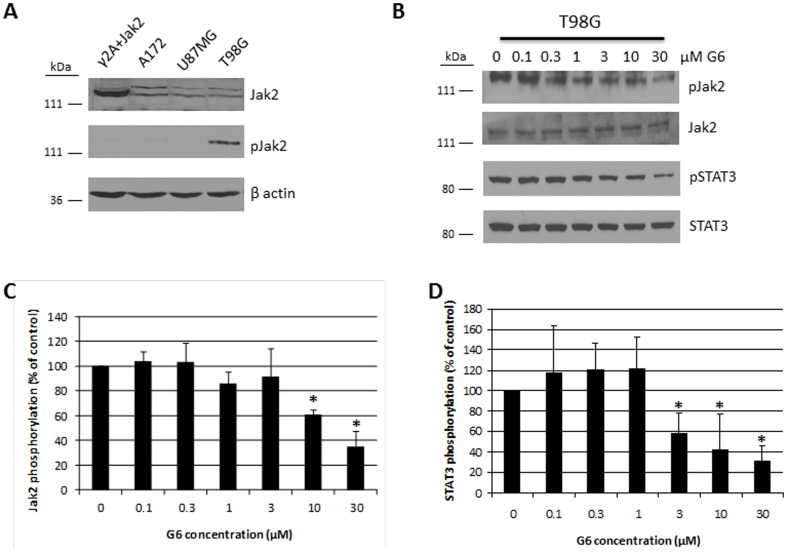
G6 reduces Jak2 and STAT3 phosphorylation in T98G cells. A) Expression of phospho-Jak2 in three glioblastoma cell lines. γ2A+Jak2 cell lysates were used as a Jak2-positive control. Shown is one of three representative blots. B) T98G cells were treated with the indicated concentrations of G6 for 24 hours and examined for Jak2 and STAT3 phosphorylation levels by Western blot. Shown is one of three representative blots for each protein. Quantification of the Jak2 (C) and STAT3 (D) phosphorylation levels (n = 3 for each). *, p<0.05 relative to DMSO control treated cells as determined by ANOVA.

Expression of active Jak2 and/or STAT3 has been observed in a number of immortalized glioblastoma cell lines and primary cells [5]–[7]. Here, we initially examined expression of Jak2 protein in three glioblastoma cell lines; namely, A172, U87MG, and T98G. Using Western blot analysis, we found that while all three cell lines expressed readily detectable levels of total Jak2 protein, only in the T98G cell line was that protein active, as indicated by the high levels of phospho-Jak2 (Figure 1A). Based on these initial screening results, we selected the T98G cell line for further experiments in testing the hypothesis that Jak2 inhibitors are efficacious against Jak2-dependent GBM tumorigenesis.

We next examined the effects of G6 treatment on Jak2 and STAT3 phosphorylation in T98G cells. For this, cells were treated for 24 hours at the indicated concentrations and we found that G6 caused a dose-dependent decrease in Jak2 and STAT3 phosphorylation levels (Figure 1B). The phosphorylation levels of these two proteins were then quantified by densitometry and normalized to total protein levels (Figures 1C and 1D). These results indicate that G6 effectively inhibits Jak2/STAT3 signaling in T98G cells. We also examined the effects of the pan-tyrosine kinase inhibitor, AG490, which is commonly used as a Jak2 inhibitor in cell-based assays. We found that AG490 modestly inhibited Jak2 phosphorylation, but in a manner that was not entirely dose-dependent, even at 100 µM (Figure S1). Thus, in addition to being a more specific Jak2 inhibitor, G6 appears to either have greater potency than AG490 or it inhibits different off-target kinases, whose inhibition may cooperate with Jak2 inhibition to elicit the effects observed with G6 treatment.

### G6 Inhibits Cell Proliferation and Colony Formation in T98G Cells

**Figure 2:**
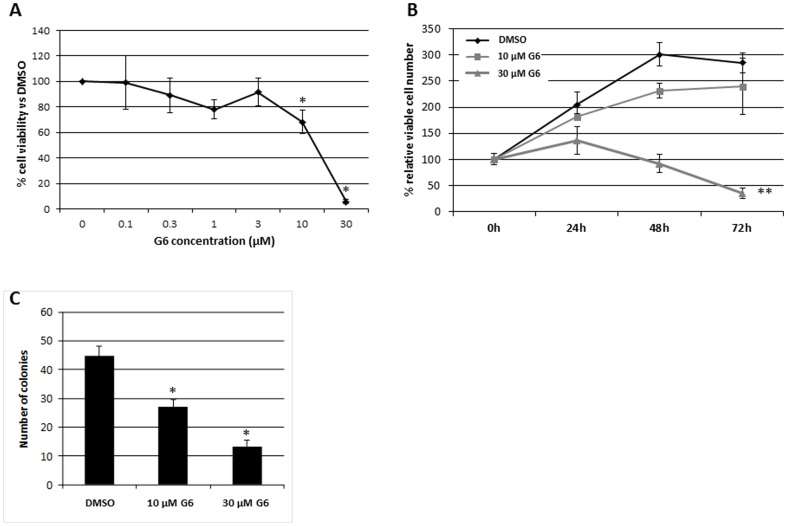
G6 treatment reduces T98G cell viability and clonogenic growth potential. A) T98G cells were treated with increasing concentrations of G6 for 72 hours and cell viability was measured by MTS assay. Each point was measured in triplicate. Shown is one of three representative results. The 10 µM and 30 µM conditions were significantly lower (p<0.05) when compared to cells treated with DMSO alone as determined by ANOVA. B) T98G cells were treated with G6 for the indicated times and concentrations. Cell viability was then measured via MTS. Each point was measured in triplicate. Shown is one of three representative results. **, p<0.01 relative to DMSO treated cells as determined by ANOVA. C) T98G cells were treated with G6 for 24 hours at the indicated concentrations, washed, and seeded in 100 mm dishes. Cells were grown for nine additional days, stained with crystal violet, and colonies were then counted. Shown is the average of four representative results. *, p<0.05 relative to DMSO treated cells as determined by ANOVA.

We next wanted to determine if the G6-dependent inhibition of Jak2 phosphorylation in T98G cells would correlate with a reduction of cell viability and/or colony formation potential. For measurements of cell viability, T98G cells were treated with increasing concentrations of G6 for the indicated times and viability was determined by the colorimetric MTS assay. We found that G6 caused both a dose- and time-dependent decrease in T98G cell viability (Figures 2A and 2B). For colony formation assays, cells were treated with the indicated concentrations of G6 for 24 hours, washed extensively with PBS, and the numbers of colonies were determined nine days later. We found that G6 caused a dose-dependent decrease in the ability of T98G cells to form colonies (Figure 2C). Collectively, the data in Figure 2 indicate that G6 reduces both the cell viability and the colony forming potential of T98G cells *in vitro*.

### G6 Reduces the Migratory and Invasive Potential of T98G Cells

**Figure 3:**
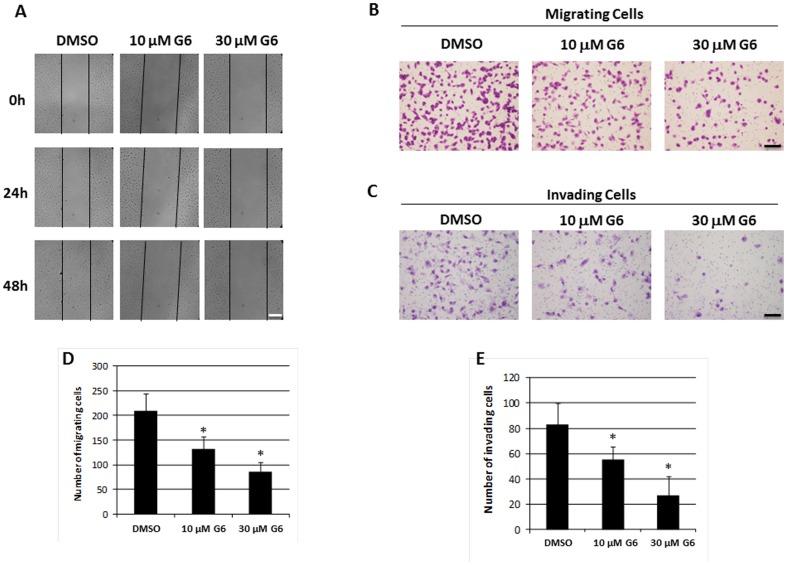
G6 reduces migratory and invasive potential of T98G cells. A) Cells were treated with G6 for 24 hours at the indicated concentrations, washed, and a scratch was made. Cell migration was monitored 24 and 48 hours later. Shown is one of three representative results. B) Cells were treated with G6 for 24 hours at the indicated concentrations, washed, and plated in un-coated 24-well inserts. Cell migration was examined by crystal violet stain after 24 hours. Shown is one of three representative results. C) Cells were treated with G6 for 24 hours at the indicated concentrations and plated in matrigel-coated 24-well inserts. Cell invasion was examined by crystal violet stain after 48 hours. Shown is one of three representative results. D) Quantification (n = 3) of migrating cells. *, p<0.05 vs. DMSO control treated cells as determined by ANOVA. E) Quantification (n = 3) of invading cells. Scale bars represent either 100 µm (A) or 50 µm (B and C). *, p<0.05 vs. DMSO control treated cells as determined by ANOVA.

The ability to migrate into and invade surrounding tissue is an important clinical characteristic of glioblastoma cells. Here, we wanted to determine if G6 treatment could reduce the migratory and/or invasive potential of T98G cells. We first used a scratch assay to examine cell migration. Cells were grown to confluency and treated with G6 for 24 hours, at which point the drug was removed, and a scratch was made. We found that G6 treatment markedly reduced the migration of cells back into the scratch area (Figure 3A). For a more quantitative examination of cell migration, we used a trans-well migration/invasion assay. Specifically, cells were treated with G6 for 24 hours and then plated in 24-well chamber inserts. Inserts were either uncoated for migration assays, or coated with matrigel for the invasion assays. The numbers of migrating and invading cells were then determined. We found that when compared to cells that were treated with DMSO control solution, G6 treatment reduced cell migration and invasion and this effect was dose-dependent (Figures 3B, 3C, 3D, and 3E). These results indicate that G6 is effective at significantly reducing both the migratory and invasive potential of T98G glioblastoma cells.

### Lentivirus-Mediated Knockdown of Jak2 Reduces Migratory and Invasive Potential of T98G Cells

**Figure 4:**
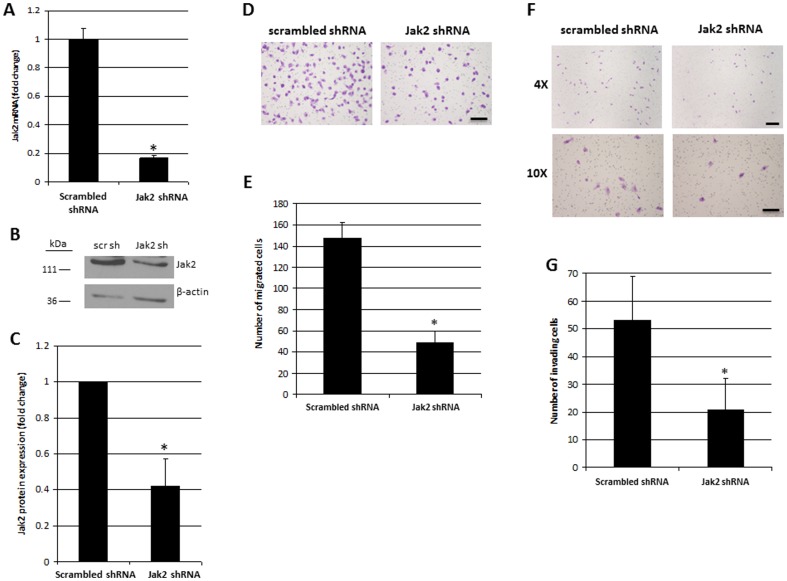
Jak2 knockdown reduces the migratory and invasive potential of T98G cells. A) Jak2 mRNA expression was examined by qRT-PCR. Each sample was measured in triplicate. Shown is one of three representative results. B) Jak2 protein expression was examined by Western blot. C) Quantification of Jak2 protein expression from three independent blots. D) Cells were plated in 24-well inserts and migration was monitored after 24 hours. E) Quantification of migrating cells from four independent experiments. F) Cells were plated in matrigel-coated 24-well inserts and invasion was monitored after 48 hours. G) Quantification of invading cells from four independent experiments. Scale bars represent either 50 µm (D and 10X F) or 100 µm (4X F) *, p<0.05 relative to scrambled shRNA as determined by Student's *t* test.

We next wanted to determine if the effects of G6 on the migratory and invasive potentials of T98G cells could be reproduced by specifically knocking down Jak2. The main goal of these experiments was to determine if the effects of G6 could be attributed directly to the inhibition of Jak2 signaling, rather than to any off target effects of the drug on other proteins. Here, we used lentiviral-mediated shRNA to silence Jak2 in T98G cells. We found that Jak2 mRNA expression was reduced by approximately 80% in Jak2 knockdown cells, when compared to cells that were transfected with control scrambled shRNA (Figure 4A). We also found that Jak2 protein expression was similarly reduced in these cells (Figures 4B and 4C). After puromycin selection, cells were plated in uncoated or matrigel-coated 24-well chamber inserts and cell migration and invasion were examined. We found that Jak2 knockdown significantly reduced the migratory (Figures 4D and 4E) and invasive (Figures 4F and 4G) potentials of these cells. When taken together, we conclude that Jak2 plays an important role in maintaining the migratory and invasive potential of these cells and the ability of G6 to reduce T98G cell migration and invasion is via its Jak2 inhibiting property.

### G6 Induces Caspase-Dependent Apoptosis in T98G Cells

**Figure 5:**
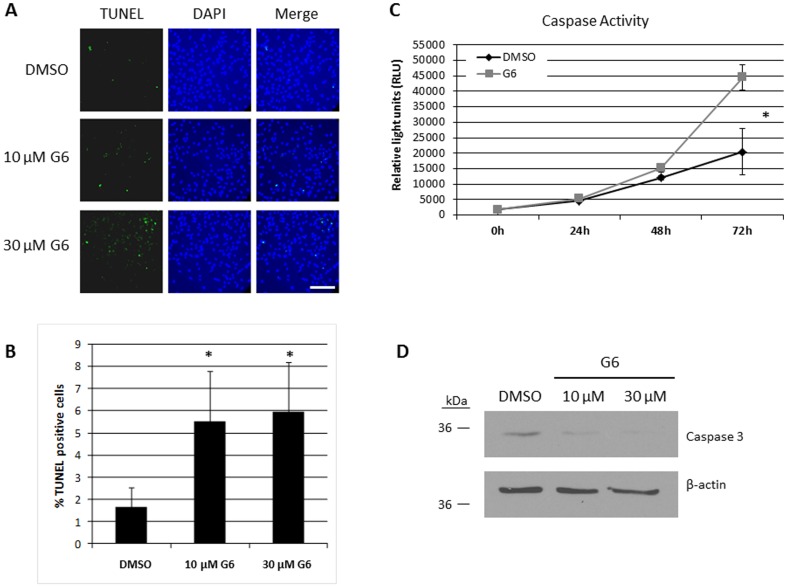
G6 induces caspase-dependent apoptosis in T98G cells. A) Cells were treated with G6 for 48 hours and apoptosis was examined by TUNEL immunofluorescence. Shown is one of three independent results. Scale bar = 50 µm. B) Quantification (n = 3) of the percentage of TUNEL-positive cells. *, p<0.05 vs. DMSO control treated cells as determined by ANOVA. C) Cells were treated for the indicated times with a 10 µM concentration of G6 and Caspase 3/7 activity was measured by a luminescent assay at the indicated times. Each point was measured in triplicate and shown is one of three independent experiments. A significant difference (*, p<0.05) was noted between the 72 hour time points as determined by ANOVA. D) Cells were treated with G6 for 48 hours and full length Caspase-3 expression was measured by Western blot. Shown is one of two representative blots.

We have previously shown that G6 induces strong caspase-dependent apoptosis in human erythroleukemia cells [14]. Therefore, we wanted to determine if this mechanism is conserved in similarly treated T98G cells. Here, T98G cells were treated with increasing concentrations of G6 for 48 hours and the levels of apoptosis were subsequently determined via TUNEL staining. We found that G6 treatment caused a dose-dependent increase in the number of TUNEL-positive cells and this was statistically significant (Figures 5A and 5B). In order to determine the specific mechanism of apoptosis in these cells, we examined the level of caspase 3/7 activity after treatment with G6. We found that G6 caused a significant increase in the level of caspase-3/7 activity when compared with DMSO control treated cells (Figure 5C). During apoptosis, full length caspase-3 is cleaved from its inactive precursor into its active form. The disappearance of full length caspase-3 typically correlates with an increase in caspase activity and thus, indicates an increase in apoptotic signaling. We examined the expression of caspase-3 by Western blot and found that G6 treatment caused a dose-dependent decrease in the levels of full-length caspase-3 (Figure 5D). Overall, these results indicate that G6 reduces T98G cell viability by inducing caspase-dependent apoptosis.

### G6 Reduces Tumor Volume in T98G Xenografts and This Is Coincident with Increased Expression of BIM and BAX

**Figure 6:**
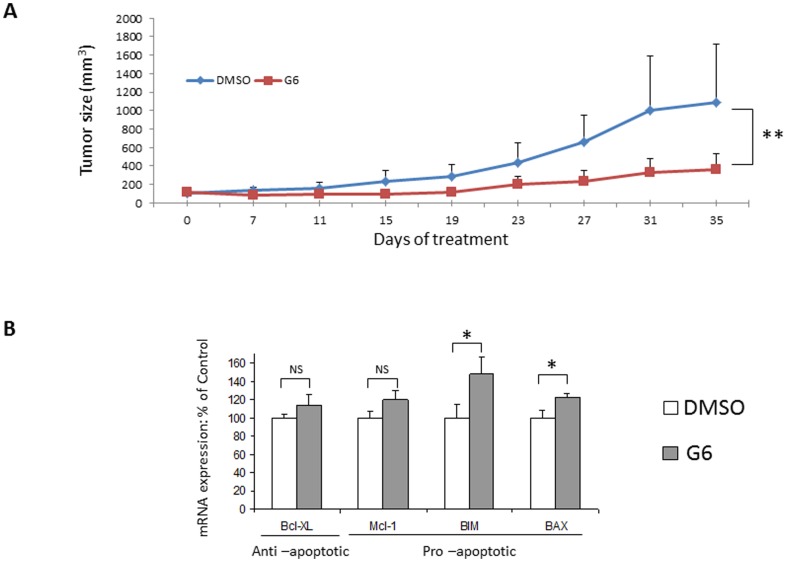
G6 reduces tumor volume in T98G xenografts and this is coincident with increased expression of BIM and BAX. A) After establishment of the tumors, mice began receiving daily injections of either DMSO (n = 4) or 10 mg/kg G6 (n = 5) at Day 0. Tumor volumes are plotted as a function of both treatment condition and time. B) Average mRNA levels of various apoptotic markers taken from the tumors. *, p<0.05, **, p<0.01.

We next tested the ability of G6 to reduce the tumorigenic potential of T98G cells *in vivo*. Specifically, T98G cells were placed in a semi-solid matrix and injected subcutaneously into the flanks of nude mice. After 90 days of growth, mice began receiving daily intraperitoneal injections of either DMSO or 10 mg/kg G6. Tumor volumes were then measured about once every five days for the ensuing 35 days, at which time the mice were euthanized. Figure 6A shows tumor volumes plotted as a function of both treatment and time. Overall, the mice that received G6 had significantly smaller tumor volumes when compared to those that received DMSO. Given the ability of G6 to induce apoptosis of T98G cells (Figure 5), a section of each tumor was immediately subjected to gene profile analysis for several different apoptotic genes. We found that while the anti-apoptotic Bcl-xL and pro-apoptotic Mcl-1 were not significantly different between the two treatment groups, mice that received G6 had significantly higher levels of pro-apoptotic BIM and BAX (Figure 6B). When taken together, these data indicate that G6 treatment reduces tumor volume, in part, via increased expression of BIM and BAX.

### Reduced Tumor Volumes Correlate with Decreased Levels of phospho-Jak2 and phospho-STAT3

**Figure 7:**
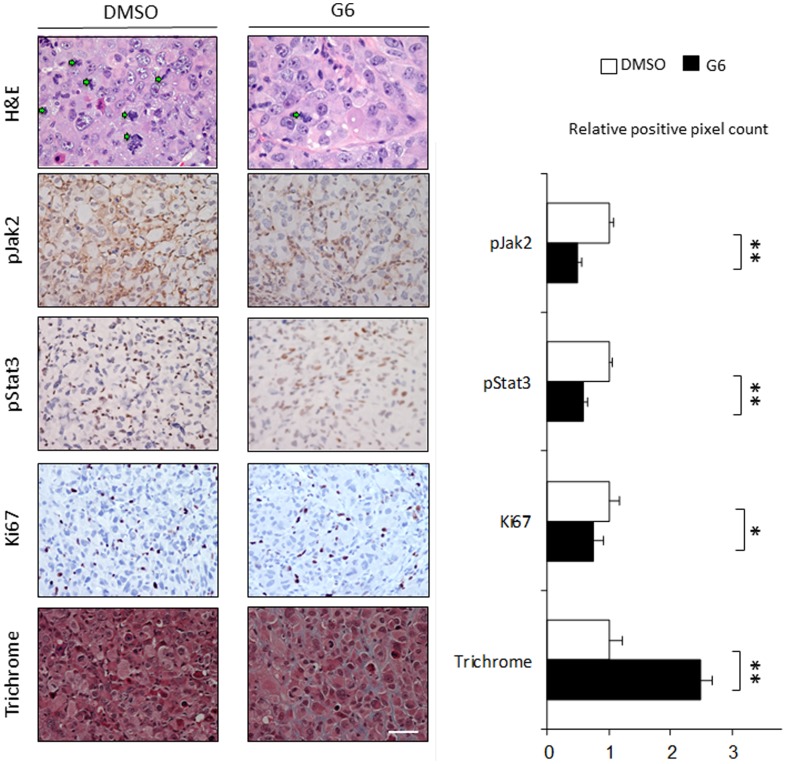
Reduced Tumor Volumes Correlate with Decreased Levels of phospho-Jak2 and phospho-STAT3. Shown are representative tumor sections, from the same nine mice described in Figure 6, after they were fixed, sectioned, and stained. For the H&E stained sections, mitotic cells are indicated by the green arrowheads. For pJak2, pSTAT3, Ki-67, and trichrome, the relative intensity of staining was quantified via relative pixel counting. Average pixel counts were then plotted as a function treatment group. The objective lens magnifications were either 50X (H&E) or 40X (pJak2, pSTAT3, Ki-67, and Trichrome). Scale bar = 50 µm. *, p<0.05, **, p<0.01.

Post mortem analyses continued with histological and immuno-histochemical examinations of the tumors. Examinations of the tumors after H&E staining found marked nuclear atypia and numerous mitotic figures in the tumors that were harvested from the DMSO treated mice, and this appeared to be reduced in the tumors that came from the G6 treated group (Figure 7). Immuno-histochemical analysis indicated that G6 reduced the levels of phospho-Jak2 and phospho-Stat3 within the tumors by an average of 58.8% and 48.6%, respectively (Figure 7). In addition to inducing discernible caspase-dependent apoptosis (Figure 5), G6 can also reduce cell proliferation [14]. To determine whether G6 had an effect on cell proliferation within the T98G derived tumors, sections were stained for Ki-67, a nuclear protein whose levels correlate directly with cell proliferation [20], [21]. We found that G6 reduced the levels of Ki-67 immuno-staining by 27% and this was statistically significant (Figure 7). Lastly, the levels of tumor fibrosis were determined via Masson's Trichrome staining. On average, the levels of fibrosis in the tumors that were harvested from G6 treated mice were >2-fold higher when compared to tumors that were taken from DMSO treated mice (Figure 7). When taken together, the data in Figure 7 indicate that treatment with G6 results in tumors that have decreased pJak2, decreased pSTAT3, decreased Ki-67, and increased fibrosis, when compared to vehicle control treated tumors.

### G6 Reduces the Levels of Vimentin Protein within T98G-Derived Tumors

**Figure 8:**
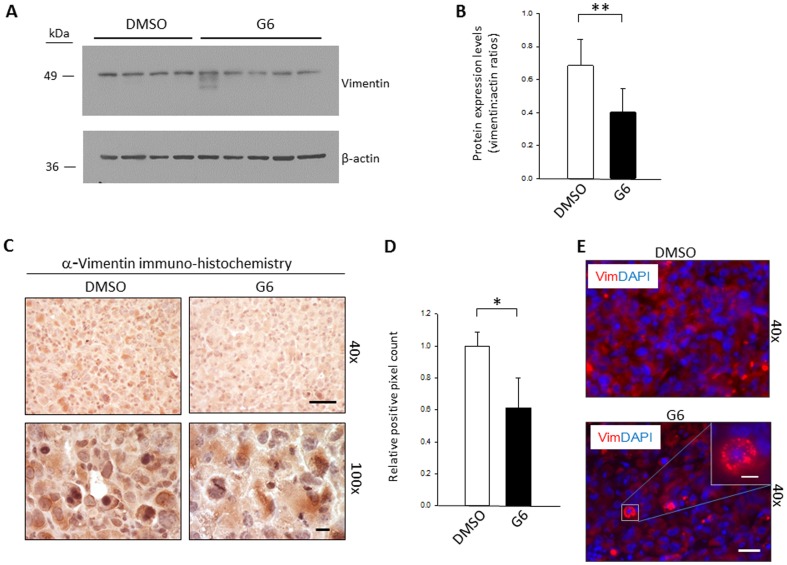
G6 Reduces the Levels of Vimentin Protein within T98G-derived Tumors. A) Expression of vimentin levels from the same nine tumor samples described in Figure 6 were determined by Western blot. Also shown are the levels of β-actin which served as a loading control. B) Quantification of vimentin protein levels normalized to β-actin. C) Representative anti-vimentin immuno-histochemistry images showing decreased vimentin expression in the G6 treated tumors. Scale bars represent either 50 µm (top panels) or 10 µm (bottom panels). D) The intensity of vimentin staining was quantified via relative pixel counting and the average pixel counts were then plotted as a function treatment group. E) Tumor sections were subjected to anti-vimentin immune-fluorescence (red) and cell nuclei were visualized via DAPI staining (blue). In response to G6, the remaining vimentin protein formed some irregular perinuclear aggregates. The scale bars represent either 20 µm (large panels) or 5 µm (single inlet panel)*, p<0.05, **, p<0.01.

As noted above, a recent report identified a direct correlation between glioma grade and vimentin expression as well as a direct correlation between increased vimentin expression and temozolomide resistance [8]. Interestingly, we have previously shown that within hematopoietic cells that have constitutive Jak2 signaling, G6 not only suppresses the aberrant Jak2 signaling, but it also decreases vimentin protein levels within these same cells, and the loss of vimentin protein *per se*, is sufficient to cause tumor cell death [(22]. Here, we sought to measure vimentin protein levels within the tumors so that we could determine if G6 similarly promotes the loss of vimentin protein within GBM. For this, a portion of each tumor was first subjected to Western blot analysis with anti-vimentin antibody (Figure 8A). We found that tumors taken from G6 treated mice had significantly reduced vimentin protein levels when compared to tumors that came from DMSO treated mice and this difference was statistically significant (Figure 8B). Next, a portion of each tumor was subjected to anti-vimentin immuno-histochemistry. For the tumors that came from G6 treated mice, we found that vimentin staining was reduced when compared to tumors that came from DMSO treated mice (Figure 8C) and this difference was significant (Figure 8D). Lastly, we have previously shown that in the absence of any drug treatment, vimentin is largely distributed over the cytoplasm [22]. However, when G6 is added to cells, not only does it decrease vimentin protein levels, but for the protein that remains in the cell, it redistributes in an irregular pattern around the perinuclear region of the cell [22]. To determine if this was similarly conserved in GBM, tumor sections were subjected to anti-vimentin immuno-fluorescence and counterstained with DAPI. For the tumors that came from DMSO treated mice, vimentin was generally expressed in the cytoplasm (Figure 8E). However, for the tumors that came from G6 treated mice, the vimentin protein formed noticeable aggregates in the perinuclear region of some cells (Figure 8E). As such, the cumulative data indicate that administration of G6 to mice reduces the levels of vimentin protein within T98G-derived xenografts. Furthermore, G6 promotes atypical aggregation of the vimentin protein around the perinuclear region of some of the cells.

## Discussion

Here, we have identified the Jak2 small molecule inhibitor G6 as a potential therapy for Jak2-dependent glioblastoma. We found that the T98G glioblastoma cell line expresses a high level of active Jak2 (ie, pJak2), and that treatment with G6 reduced Jak2/STAT3 phosphorylation in a dose-dependent manner. We also found that this correlated with reductions in cell viability, colony forming abilities, cell migration, and cell invasion properties. The ability of G6 to promote these beneficial effects appears to be via its ability to inhibit Jak2, rather than via any off target effects as some of these results were recapitulated via the specific knock down of Jak2 by shRNA. Lastly, when examined *in vivo*, we found that G6 treatment significantly reduced tumor volumes. Furthermore, tumors that were harvested from G6 treated mice exhibited increased apoptosis, decreased pJak2, decreased pSTAT3, decreased Ki-67, increased fibrosis, and decreased vimentin levels when compared to vehicle control treated mice. Collectively, these results indicate that G6 has discernible therapeutic efficacy in glioblastoma cells that express active Jak2.

In the initial studies examining the role of Jak2 in glioblastoma, the tyrphostin compound, AG490, was used to block Jak2 activation. These studies demonstrated that inhibition of tyrosine kinases in general, and Jak2 in particular, could impact the viability and invasive properties of GBM cells [9], [23]. Our results contained herein provide additional evidence of a role for Jak2 signaling in glioblastoma cells and the potential application of Jak2 inhibitors for GBM therapy. Interestingly however, in a set of studies where we examined the effect of G6 on U87MG cells, a GBM cell line where Jak2 is not hyper-phosphorylated, G6 was not as efficacious as it was in T98G cells (Figure S2). Along similar lines, in a very recent Phase I clinical study, the Jak2 inhibitor AZD1480 was given as a monotherapy to 38 patients with advanced solid tumors, with none being GBM. In addition, no consideration was given as to the status of the Jak/STAT signaling pathway in these patients. The authors found that the drug provided no observable clinical benefit, and along with a number of reported serious adverse events, this has led to an abandonment of the study [24]. Thus, the success of Jak2 inhibitor therapy for GBM, or any solid tumor for that matter, may first require the identification of those that have dys-regulated Jak2 signaling.

The role of Jak2 signaling in glioblastoma tumors has only recently been investigated, so the clinical significance of Jak2 inhibition is still unclear. However, recent studies have demonstrated that Jak/STAT signaling is an important contributor to tumorigenicity of human glioma cells, and that specific inhibition of this pathway reduces tumorigenic potential of these cells [25]–[26]. One of these studies utilized derivatives of AG490 to effectively target brain tumor stem cells (BTSCs) in a xenograft model [26]. In addition, another recent study showed that Jak2/STAT3 signaling is essential in the migration, invasion, and tumor progression of EGFRvIII glioblastoma cell lines [27]. Moreover, this study revealed that only Jak2 inhibition, not STAT3 nor EGFR knockdown, was able to suppress the migratory and invasive phenotype of these EGFR mutant cell lines. In another study, it was found that an allosteric Jak2 inhibitor had greater efficacy against EGFR vIII-expressing cell lines than wild-type EGFR cells [28]. EGFRvIII mutations are observed in a large subset of glioblastoma tumors, including 20% of newly diagnosed glioblastoma patients in a recent study [29] and up to 67% in previous patient cohorts [30]. Given the high occurrence of this mutation and the selectivity of Jak2 inhibitors for EGFRvIII-positive glioma cells, stratifying patients by EGFR mutation status may be an important factor in assigning Jak2 inhibitor therapy.

In order for G6 to have a potential role in the treatment of GBM, it must cross the blood-brain barrier and reach therapeutic concentrations within this organ. To determine if this was possible, naïve mice were intravenously injected with G6 and drug concentrations were subsequently determined in the brain. We found that G6 could readily cross the blood-brain barrier and could maintain IC_50_ inhibitory values for at least six hours after drug administration (unpublished observations). As such, these results indicate that G6 can potentially target GBM or other central nervous system derived diseases.

With respect to the tumorigenic growth potential of T98G cells, we found that G6 treatment reduced pJak2, pSTAT3, cell proliferation, cell migration, cell invasion, and induced apoptosis. While these events are all highly desirable for any chemotherapeutic agent, a clear yet less understood phenomenon that we repeatedly observed was that G6 treatment lead to increased levels of fibrosis within the tumors themselves. Interestingly, examination of the lungs, livers, and kidneys from these same animals found no fibrosis whatsoever, suggesting that the increased fibrosis in the G6 treated mice was a tumor-specific event. Consistent with this observation that G6 does not promote host organ fibrosis is a previous work where we reported that a 30 day administration of G6 into C57BL/6 mice did not produce any histological abnormalities, such as fibrosis, within the hearts, brains, kidneys, or lungs of the treated animals [14]. However, fibrosis within host tissues and tumor tissue is quite different and it is still unclear whether increased tumor fibrosis is advantageous or deleterious to the survival of the animal [31], [32]. What is clear, however, is that the tumors from G6 treated mice had noticeably increased levels of collagen deposition when compared to mice that received DMSO. This is an area of current investigation as we are trying to determine whether the G6-mediated tumor fibrosis is due to the expansion of fibroblasts subsequent to the death of T98G cells within the tumor, alterations in the epithelial-mesenchymal transition of cells by G6, fibrotic re-modeling that is subsequent to the resolution of an inflammatory response within the tumor, or yet another mechanism. Additionally, it will be of interest to see if other Jak2 inhibitors produce a similar type fibrotic effect in GBM tumor models.

Another significant component of this work is the observation that G6 treatment reduces the levels of vimentin within the T98G-derived tumors. Within solid tumors in general, vimentin expression correlates with accelerated tumor growth, increased metastatic potential, and poorer prognosis [6]. Within brain tumors in particular, vimentin expression also correlates with temozolomide resistance, a frontline therapy for the treatment of GBM [8]. Presumably, a treatment that reduces vimentin expression, such as G6, may result in decreased tumor growth, decreased metastatic potential, increased sensitivity to temozolomide, and improved patient prognosis. Thus, in addition to being used as a monotherapy as we demonstrated here, G6 may have potential as an adjunct therapy with other agents such as temozolomide for the treatment of GBM. It has recently been suggested that GBM tumors can be subcategorized into three classes based on expression levels of intermediate filament proteins, including vimentin [33]. Therefore, stratification of patients based on vimentin expression and Jak2 activation status may eventually allow for maximal therapeutic efficacy of Jak2 inhibitor therapy in GBM.

Cancers in general and gliomas in particular are characterized by a number of cellular perturbations including increased cell division, avoidance of apoptosis, enhanced clonogenic growth potentials, enhanced migratory potentials, enhanced invasive potentials, and an ability to promote tumor vascularization. Chemotherapeutic agents generally target 1–2 of these properties and this of course provides the rational basis for combination drug therapies for the treatment of cancer. In the case of G6 however, we found that it was efficacious for a multitude of cellular responses including proliferation, apoptosis, migration, invasion, clonogenic growth, and perhaps even a de-vascularization effect. These pleiotropic effects of G6 are consistent with the known roles of Jak2 in these same biological processes [34]. Thus, because of its role in a number of processes that are critical for tumor maintenance and growth, Jak2 inhibitor therapy may be more efficacious than other targeted therapies for tumors in which Jak2 signaling is dys-regulated.

In summary, we found that the T98G cell line expressed readily detectable levels of active Jak2 protein. We found that G6 treatment of these cells significantly reduced their tumorigenic potential both *in vitro* and *in vivo*. When taken together, our data indicate that Jak2 inhibitors in general, and G6 in particular, may be viable therapeutic options against GBM exhibiting constitutive Jak2 signaling.

## Supporting Information

Figure S1 **Treatment of T98G Cells with AG490.** T98G cells were seeded in 100 mm dishes and treated with the indicated concentrations of AG490 for 24 hours. Soluble protein lysates were then prepared and Jak2 was immuno-precipitated from the lysates via the addition of anti-Jak2 antibody. After separation by SDS-PAGE, the samples were Western blotted with either anti-pJak2 (top) or anti-Jak2 (bottom) antibody.
(TIF)

Figure S2 **Effect of G6 on U87MG Cell Viability.** U87MG cells were seeded in 96-well plates and then treated with the indicated concentrations of G6. 72 hours later, cell viability was determined via MTS. Each point was measured in triplicate. Shown are the average number of cells (mean +/− SD) normalized to cells that received DMSO alone.
(TIF)
