# Supplementary figures and images for: The Jak2 Small Molecule Inhibitor, G6, Reduces the Tumorigenic Potential of T98G Glioblastoma Cells In Vitro and In Vivo
Source: PLoS One. 2014 Aug 27;9(8):e105568. doi: 10.1371/journal.pone.0105568 (PMC4146502; doi:10.1371/journal.pone.0105568)

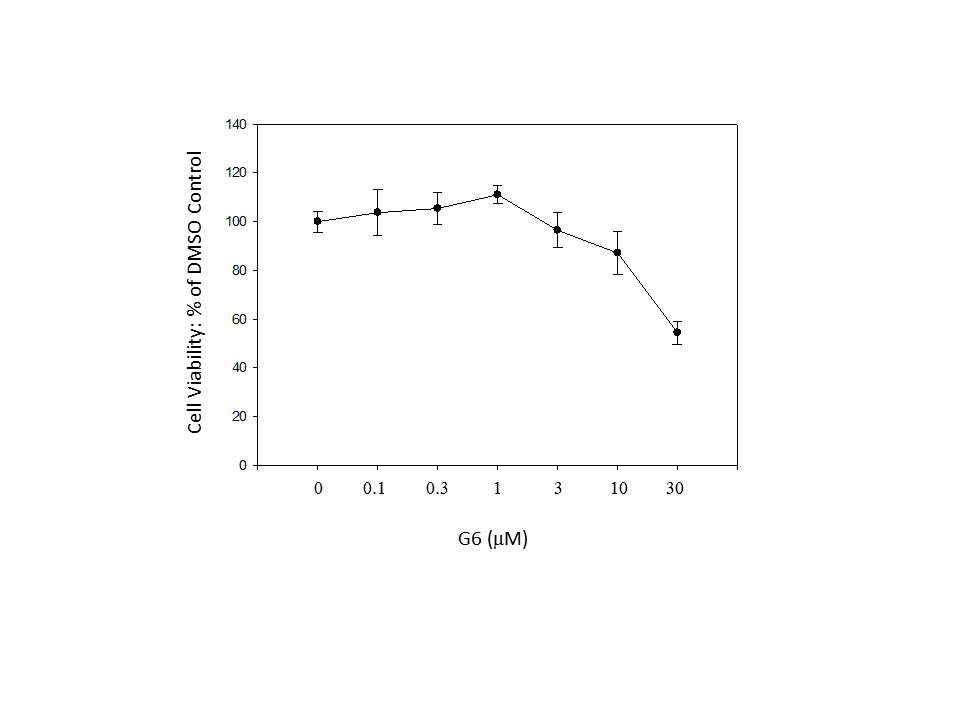

Supplement: Figure S2 — Effect of G6 on U87MG Cell Viability. U87MG cells were seeded in 96-well plates and then treated with the indicated concentrations of G6. 72 hours later, cell viability was determined via MTS. Each point was measured in triplicate. Shown are the average number of cells (mean +/− SD) normalized to cells that received DMSO alone. (TIF) [file pone.0105568.s002.tif]
